# Research progress and challenges of stem cell therapy for ischemic stroke

**DOI:** 10.3389/fcell.2024.1410732

**Published:** 2024-07-08

**Authors:** Zaihong Cha, Yisheng Qiao, Qixiong Lu, Qiyang Wang, Xiaoyang Lu, Hu Zhou, Tao Li

**Affiliations:** ^1^ The Affiliated Hospital of Kunming University of Science and Technology, Kunming, Yunnan, China; ^2^ Department of Orthopedics, The First People’s Hospital of Yunnan Province, The Affiliated Hospital of Kunming University of Science and Technology, Kunming, Yunnan, China; ^3^ Department of Neurosurgery, The First People’s Hospital of Yunnan Province, The Affiliated Hospital of Kunming University of Science and Technology, Kunming, Yunnan, China; ^4^ Research Center for Clinical Medicine, The First People’s Hospital of Yunnan Province, The Affiliated Hospital of Kunming University of Science and Technology, Kunming, Yunnan, China; ^5^ Institute of Neurosurgery and Neuroscience, The First People’s Hospital of Yunnan Province, The Affiliated Hospital of Kunming University of Science and Technology, Kunming, Yunnan, China

**Keywords:** ischemic stroke, stem cells, immunomodulation, exosome, autophagy, microbiota-gut-brain axis

## Abstract

Ischemic stroke is a significant global cause of death and disability. Currently, treatment options for acute ischemic stroke are limited to intravenous thrombolysis and mechanical recanalization. Therefore, novel neuroprotective strategies are imperative. Stem cell transplantation possesses the capabilities of differentiation, proliferation, neuronal replacement, nerve pathway reconstruction, secretion of nerve growth factors, and enhancement of the microenvironment; thus, it is a potential therapeutic approach for ischemic stroke. In addition, the immunomodulatory function of stem cells and the combined treatment of stem cells and exosomes exhibit a favorable protective effect on brain injury and neurological dysfunction following stroke. Meanwhile, the theory of microbiota-gut-brain axis provides us with a novel perspective for comprehending and managing neurological diseases. Lastly, stem cell transplantation has demonstrated promising outcomes not only in treating ischemic stroke but also in dealing with other neurological disorders, such as brain tumors. Furthermore, challenges related to the tissue source, delivery method, immune response, and timing of transplantation still need to be addressed to optimize the treatment.

## 1 Introduction

**FIGURE 1:**
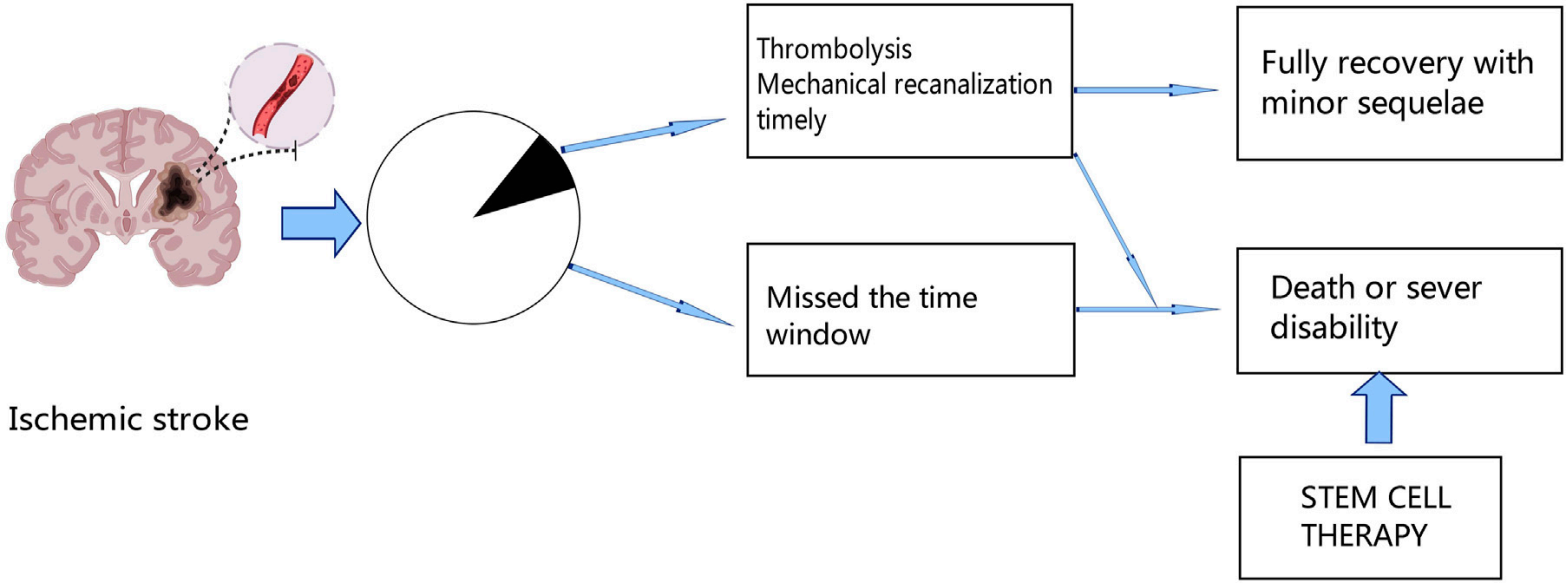
After ischemic stroke, only a fraction of patients can receive thrombolytic therapy or mechanical recanalization therapy in time, and a small proportion of these patients can fully recover with minor sequelae, while the rest of the patients who miss the treatment time window will die or remain functional disability. Thus, stem cell therapy offers new hope for the future.

In the world, stroke is a leading cause of mortality and permanent disability; ischemic stroke makes up over 80% of all stroke cases (Saini et al., 2021). Ischemic stroke has had a considerable negative impact on human society, particularly in Asia and Africa (Ding et al., 2022). Even though there are several treatment options, including mechanical thrombectomy and chemical thrombolysis with recombinant tissue plasminogen activator (tPA) (Campbell and Khatri, 2020; Rabinstein, 2020), many patients will have missed the best time to receive treatment due to the restricted window, which can lead to severe neurological deficits. Therefore, to improve the outcome of ischemic stroke, new therapies are urgently needed Figure 1.

The effectiveness of stem cells in treating ischemic stroke has attracted more and more attention in recent years (Zakrzewski et al., 2019; Shehjar et al., 2023). Currently, mesenchymal stem cells (MSCs), embryonic stem cells (ESCs), and neural stem cells (NSCs) are the three primary types of stem cells employed in stroke therapy (Mosconi and Paciaroni, 2022). MSCs are superior to other cell types in some respects. Firstly, MSCs can be conveniently isolated from bone marrow, adipose tissue, umbilical cord blood and other tissues, and can be easily cultured (Ding et al., 2011). Secondly, MSCs have immunomodulatory properties that reduce the risk of rejection and transplantation complications (Ankrum et al., 2014), which makes them a favorable cell type for allogeneic transplantation. Thirdly, the use of MSCs delicately avoids the ethical issues that NSCs or ESCs provoke. However, it is worth to note that MSCs from different sources are heterogeneous, and thus standardized methods are problematic to establish to ensure the quality and consistency of cells used in research or clinical practice (Wang and Han, 2019). NSCs can differentiate into functional neurons and glial cells and are theoretically the best choice for central nervous system (CNS) recovery (Ottoboni et al., 2017; Zhao T. et al., 2022). Despite this, their application is limited by a variety of factors, such as the infeasibility in obtaining the cells from matured tissue, limited number of cells, problems of *in vitro* culture (Nam et al., 2015; Fernandez-Munoz et al., 2021). ESCs have self-renewal and pluripotency, and can differentiate into all types of cells except placenta (Hughes et al., 2011; Varzideh et al., 2023). They have a broad prospect in medicine, but their practical application will be limited by ethics (de Miguel-Beriain, 2015), and there is a potential risk of malignant transformation and teratoma formation (Nouspikel, 2013).

**FIGURE 2:**
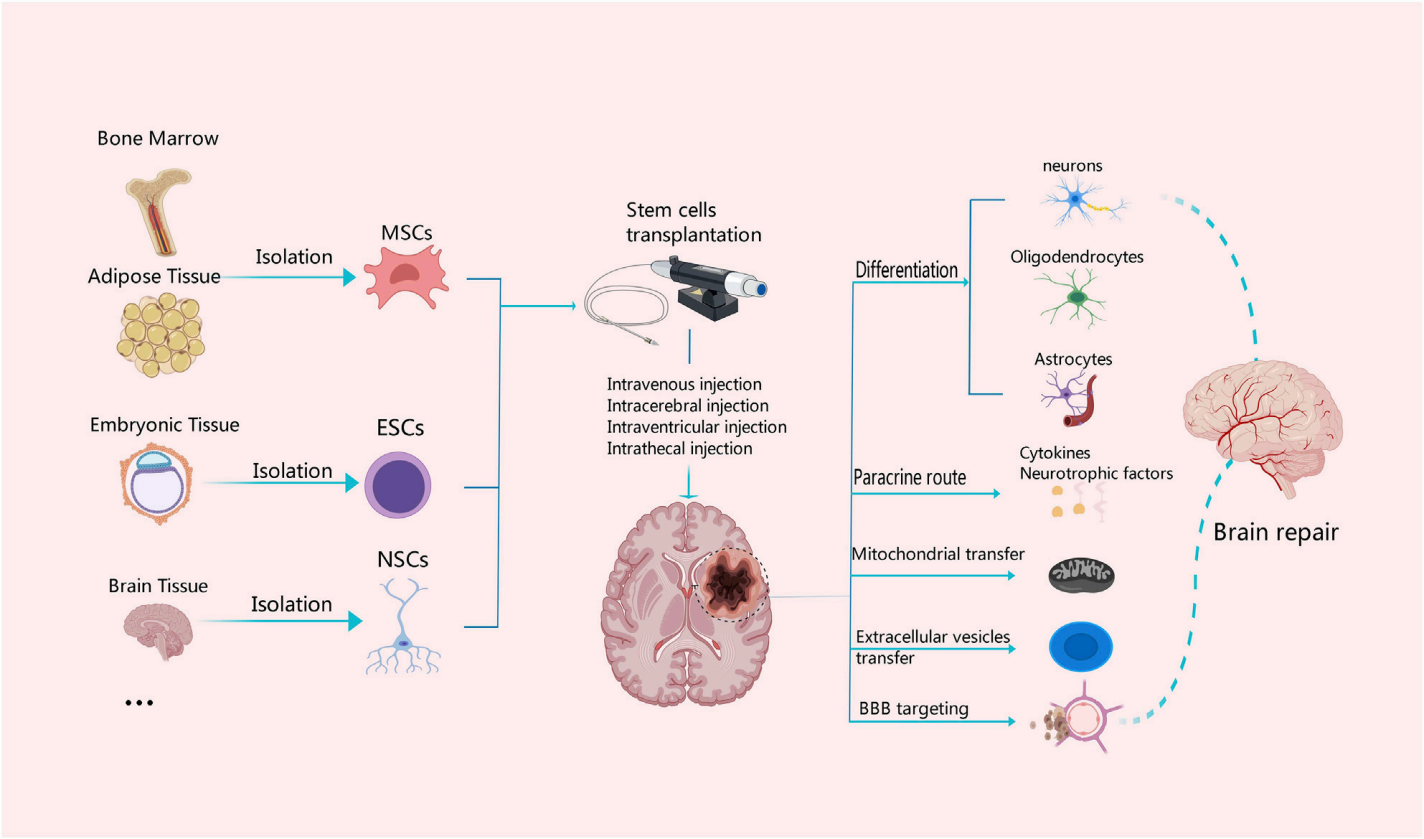
Mechanisms associated with different tissue-derived stem cells in the treatment of ischemic stroke. MSCs can be derived from bone marrow and adipose tissue, ESCs from embryonic tissue, and NSCs from the central nervous system. Stem cells from different tissue sources can be transplanted with intravenous or arterial injection or other measures. Stem cells have the capability of multi-directional differentiation; they can differentiate into neurons, oligodendrocytes and astrocytes, and secrete cytokines and neurotrophic factors, as well as promote the BBB reconstruction and neuronal repair through diverse approaches such as mitochondrial transfer and secretion of extracellular vesicles.

Stem cells have the ability to differentiate into various cell types, including mature neurons, oligodendrocytes and astrocytes Figure 2. They also release a multitude of neurotrophic factors and cytokines that can facilitate neurogenesis, replace damaged cells, and promote nerve function recovery (Monica Chau et al., 2016; Chrostek et al., 2019; Zhou et al., 2021). Stem cells secrete vascular endothelial growth factor (VEGF), basic fibroblast growth factor (bFGF), and placental growth factor (PLGF), which contribute to angiogenesis, repair of the vascular network, as well as improvement in blood supply and oxygen delivery to the brain (Fang et al., 2023). By activating mechanisms involved in mitochondrial quality control, such as mitochondrial division and fusion, mitochondrial autophagy, mitochondrial biogenesis, and intercellular mitochondrial transfer, stem cells help preserve the integrity and functionality of the mitochondrial network. This preservation aids in enhancing the microenvironment for nerve regeneration while reducing inflammation (Pickles et al., 2018; An et al., 2021). In addition, the pathogenesis of ischemic stroke is closely related to inflammation and abnormal activation of the immune system. Stem cells have the potential to combat inflammation and restore immune system balance through paracrine signaling pathways and regulation of autophagy (He et al., 2021a; Tan et al., 2022). Combining stem cells and exosomes demonstrates promising efficacy in mitigating brain injury and ameliorating neurological dysfunction following stroke. As a nanotherapeutic agent, exosomes derived from stem cells can deliver growth factors, proteins, nucleic acids, and microRNAs (miRNAs) into target cells via fusion with their membranes, thereby modulating the function and activity of these target cells (Gurunathan et al., 2021). Additionally, the theory of the microbiota-gut-brain axis offers us a novel perspective to comprehend and address neurological disorders. By modulating the intestinal microbial community, enhancing intestinal immune function, regulating the neuroendocrine system, and activating neurotrophic factors (Chidambaram et al., 2022), the microbiota-gut-brain axis can enhance the self-repair capacity of the nervous system. Furthermore, the immunomodulatory function of stem cells not only plays a crucial role in the treatment of ischemic stroke but also has significant potential to improve the immunotherapy effect of glioblastoma in the future (Shah, 2016; Fan et al., 2023). The efficacy of stem cells in the treatment of ischemic stroke has been demonstrated by multiple preclinical and clinical studies with positive results. Stem cell transplantation has been shown in animal experiments to drastically ameliorate neurological impairments, increase neurogenesis, and reduce infarct size in ischemic brains (Chen et al., 2001; Yang et al., 2022). Also, stem cell treatment is safe and feasible in patients who have had an ischemic stroke in early clinical trials. Some studies have even reported improvements in functional outcomes and quality of life (Chen et al., 2014; Qiao et al., 2014; Steinberg et al., 2016; Chung et al., 2021). However, several challenges and limitations need to be addressed to advance the clinical translation of stem cell therapy for ischemic stroke. One of the major challenges is to optimize the delivery, survival, and integration of transplanted stem cells into the ischemic brain tissue. Strategies to enhance cell engraftment and promote long-term survival are critical for maximizing the therapeutic potential of stem cell therapy (Lee et al., 2008; Savitz et al., 2011; Stonesifer et al., 2017). Additionally, the heterogeneity of stroke patients, variability in stroke severity and etiology, and differences in treatment protocols pose challenges for standardizing stem cell-based therapies and evaluating their efficacy across diverse patient populations (Boncoraglio et al., 2019; Cui et al., 2019).

In recent years, there has been extensive research on the role of stem cells in acute stroke. Most studies have demonstrated that stem cells mitigate nerve damage and exert a neuroprotective effect through various mechanisms. This article provides an overview of the involvement of stem cells in ischemic stroke and suggests their potential as interventions for managing and treating it. Additionally, it discusses the neuroprotective impact of the microbiota-gut-brain axis and its recent advancements, while also proposing new prospects for refractory intracranial tumor treatment.

## 2 Ischemic stroke

The occurrence of ischemic stroke is the neuronal death and functional impairment caused by insufficient blood supply to the brain (Feske, 2021). Hypoxia and a lack of glucose in the brain tissue stimulate anaerobic glycolysis, lactic acid buildup and acidosis. These processes result into malfunctioning mitochondria and intracellular Ca^2+^ overload in brain cells, which stops the synthesis of adenosine triphosphate (ATP) and produces excessive reactive oxygen species (ROS) (An et al., 2021). ATP is essential for generating an electrochemical gradient across the plasma membrane and for synaptic transmission in the nervous system (Li and Sheng, 2022). In addition, the over-production of ROS in the body may cause oxidative stress (OS), leading to oxidative damage of cell membrane, proteins, nucleic acids and other biomolecules in brain cells. It is an alleged ischemic injury cascade, including inflammatory responses, cell apoptosis, autophagy, and necrosis (Orellana-Urzua et al., 2020; Qin et al., 2022). The production of TNF-α, IL-6, IL-12 and other inflammatory cytokines can also break down the blood-brain barrier (BBB), allowing blood-derived substances to pass through and induce hemorrhagic transformation and vasogenic edema. This makes a way for peripheral inflammatory cells to enter the brain parenchyma, which can result in neuronal death and increase patient mortality. Microglia and astrocytes in the ischemic zone sustain comparable damage to neurons (Yang et al., 2019; Gao et al., 2023). A prospective tactic for tissue regeneration and neuroprotection has therefore developed the focusing on these processes.

## 3 Mechanisms of stem cell therapy

### 3.1 Promotion of neuroprotection and regeneration

The survival of neurons profoundly impacts the stability and integrity of brain function. Enhancing the protection of neurons and promoting the neuronal repair and regeneration have been the main focus of effective treatment of brain injury (Zhao Y. et al., 2022). A multitude of neurotrophic factors and cytokines, including VEGF, brain-derived neurotropic factor (BNDF), bFGF, nerve growth factor (NGF) and insulin-like growth factor-1 (IGF-1) are released by stem cells upon differentiating into different cell types that form neural tissue (Kaminska et al., 2022) Figure 2. These neurotrophic factors and cytokines help promote the replacement of damaged cells, neurogenesis, and functional recovery of brain tissues. Simultaneously, the released cytokines and neurotrophic factors can activate neuroprotective pathways, shield neurons from more damage, prevent neuronal death, and increase the ability of surviving neurons to regenerate their axons and regain activity (Palma-Tortosa et al., 2021; Haupt et al., 2023). Lin et al. have identified a nanoparticle that can induce stem cell differentiation by delivering superparamagnetic iron oxide (SPIO) nanoparticles and small interfering RNA/antisense oligonucleotides (siRNA/ASO) to NSCs and can also track NSCs *in vivo* using magnetic resonance imaging (Lin et al., 2021). Furthermore, Song et al. also found targeting extracellular vesicles derived from M2-microglia (M2-microglia–derived small extracellular vesicles [M2-sEVs]) is beneficial for CNS repair, as M2-sEVs carry a significant amount of microRNA-124 (miR-124), which promotes functional recovery after ischemic stroke by enhancing the proliferation and differentiation of NSCs (Song et al., 2023). Because NSCs have the ability to self-renew and differentiate into several types of cells in the CNS, as well as the advantages of low immunogenicity, autologous extraction, easy *in vitro* culture, and rapid proliferation. Thus, NSCs transplantation have the most significant favorable effects in promoting nerve regeneration and repair (Zhao T. et al., 2022; Tang et al., 2023). Ryu S et al. demonstrated experimentally that human neural stem cells (hNSCs) can increase the proliferation and angiogenesis of endogenous NSCs, reduce the volume of cerebral infarction, and promote functional recovery in rats with cerebral ischemia (Ryu et al., 2016). Furthermore, a study by Zhang et al. found a significant recovery of neurological function in rats after bone marrow mesenchymal stem cells (BMSCs) transplantation, which was associated with the downregulation of the inhibitory factors Nogo-A, NgR, and RhoA (Zhang et al., 2020). In addition, Afsaneh Asgari Taei et al. showed that conditioned medium (CM) derived from human embryonic stem cells-MSCs (hESCs-MSCs) can accelerate the recovery from cerebral ischemic injury by improving neural regeneration and angiogenesis (Asgari Taei et al., 2021). Therefore, the treatment of stroke may not require direct transplantation of stem cells. The secretome of MSCs generated from various tissues has a varied makeup. Research findings indicated that placental-MSCs released more hepatocyte growth factor (HGF) and prostaglandin E2 (PGE2), whereas BMSCs secreted more VEGF. Adipose-derived MSCs express higher levels of VEGF-D, IGF-1 and IL-8, while dermal-derived MSCs secrete more leptin (Asgari Taei et al., 2022).

### 3.2 Reestablishment of blood supply and repair of the BBB

Not only must damaged nerve tissue be repaired, but arteries must be re-opened to treat stroke. Microangiogenesis promotes cerebral blood flow and nutrient supply to the traumatic area (Moon et al., 2021). Numerous protein factors including VEGF, bFGF and govern angiogenesis and vascular maturation (Rosell-Novel et al., 2004). Additionally, stem cells could develop into extracellular matrix and vascular endothelial cells, create new blood vessels, repair the vascular network and enhance blood flow, all of which improve the oxygen and blood supply to the brain (Fang et al., 2023). LeBlanc et al. demonstrated that VEGF-B/VEGFR-1 signaling is essential for mediating neurovascular healing and confirmed that VEGF-B is a safe approach to promote the development of brain microvessels in injured areas (Jean LeBlanc et al., 2018). Bi et al. in a study using BMSCs transplantation to treat cerebral ischemia in rats with middle cerebral artery occlusion (MCAO); it was found that in addition to promoting angiogenesis by up-regulating VEGF and GFAP expression in the ischemic region, BMSCs may also have brain-protective effects (Bi et al., 2018). Nevertheless, both exogenous and endogenous VEGF trigger the breakdown of the endothelial barrier during acute cerebral ischemia (Hu Y. et al., 2022). Therefore, it is essential for determining when to provide stem cell transplantation because of VEGF’s dual function. In addition, some miRNAs, such as miR-15a/16–1 cluster have significant effects on cerebral angiogenesis. Sun et al. showed that endothelial miR-15a/16–1 cluster has a negative regulatory effect on long-term brain healing and cerebral angiogenesis after cerebral ischemia (Sun P. et al., 2020). Future research on the promotion of angiogenesis by stem cell transplantation could be developed in this direction.

The BBB is situated at the interface between the circulatory system and the CNS, serving as a physical and metabolic barricade that plays a crucial role in maintaining homeostasis within the microenvironment of the CNS (Huang et al., 2020; Zhang Y. et al., 2023). Following an ischemic stroke, disruption of the BBB is a significant pathophysiological mechanism that exacerbates brain tissue damage. It involves neutrophil infiltration, matrix metalloproteinase-9 (MMP-9) activation, tight junction (TJ) degradation, and neuroinflammatory response (Persidsky et al., 2006; Yang et al., 2007). The investigation of MSCs in the management of ischemia-induced disruption of the BBB unveils novel therapeutic targets for cerebrovascular diseases. The transplantation of MSCs can effectively maintain the integrity of the BBB following ischemic stroke (Do et al., 2021) Figure 2. Phuong Thao Do et al. found that MSC-FGF21 transplantation blocked MCAO-induced MMP-9 activation and neuroinflammation, significantly preserved BBB integrity, and improved neurological function recovery in rats with ischemic stroke (Do et al., 2023). Zhang et al. found that MSCs inhibited the expression of MMP-9 by down-regulating intercellular adhesion molecule-1 (ICAM-1) and alleviated the destruction of the blood-brain barrier in mice after ischemia (Cheng et al., 2018). Pallab Bhattacharya et al. found that MSCs treatment after stroke could not only regulate aquaporin 4 (AQP4) by regulating protein kinase Cδ (PKCδ) expression, reduce brain edema and promote neuroprotection, but also prevent further BBB disruption and favor the functional reconstruction of BBB (Datta et al., 2022). Additionally, endothelial cells of neovascularization in the peri-infarct area exhibit abnormally high BBB permeability due to the lack of tight junction protein (TJP) after stroke. TJP can promote rapid angiogenesis after stroke and re-establish a functional BBB, thereby promoting the prognosis and rehabilitation of stroke patients (Yang and Torbey, 2020). This may be an intervention point for vascular remodeling and BBB recovery after stroke.

### 3.3 Maintenance of the integrity of mitochondrial function

Apart from being crucial for the production of cellular energy and signal transduction, mitochondria are also involved in a number of other cellular functions, such as Ca^2+^ homeostasis, the generation of ROS, mitochondrial permeability transition pore (MPTP) induction, apoptosis and the inflammatory response (Yang et al., 2018; He et al., 2020). Due to the disruption of cerebral blood flow in acute ischemic stroke, the supply of oxygen and glucose is impaired, leading to cellular bioenergetic stress and failure of mitochondrial oxidative phosphorylation, as well as increased production of ROS and excessive accumulation of calcium. Finally, neurons will suffer irreversible damage (Fuhrmann and Brune, 2017; Li et al., 2022). Based on this, maintaining the integrity of mitochondrial function has become one of the topics in developing stem cell therapy for ischemic stroke.

In response to cell stress, mitochondria can activate quality control mechanisms such as mitophagy, mitochondrial fission and fusion, mitochondrial biogenesis, and mitochondrial transcellular translocation. By stabilizing the integrity and functionality of the mitochondrial network, these adaptive response mechanisms aid in restoring the homeostasis of neurovascular units (Pickles et al., 2018; An et al., 2021). In particular, damaged or dysfunctional mitochondria can be eliminated through the process of mitophagy. Mitophagy which is facilitated by the MPTP, establishes connections among intricate mitochondrial networks and influences the dynamic processes of fusion and division within these organelles. This mechanism effectively reduces ROS production in mitochondria, thereby preventing cell apoptosis. It plays a crucial role during the pathophysiological stages of cerebral ischemia (Tang et al., 2016; Guan et al., 2018; Shen et al., 2021). He et al. found that the JNK1/2-BNIP3 signaling pathway is closely related to hypoxia and can regulate mitophagy, which would be a promising target for the treatment of stroke (He et al., 2022). Nevertheless, due to the intricate nature of the mitophagy process (Zhang L. et al., 2021), there remains an ongoing debate regarding whether targeting autophagy would be more effective in disease treatment. Stem cells are capable of transferring healthy mitochondria to damaged cells through the mechanisms of tunneling nanotubes (TNTs), vesicular transfer or cell fusion Figure 2. Mitochondrial reuptake helps abolish normal cellular metabolism, prevents oxidative stress and apoptosis, and enhances neural activity, axonal regeneration and other functional recovery (Hayakawa et al., 2016; Borlongan et al., 2018). Anisha D'Souza et al. found that homotypic endothelium-derived extracellular vesicles (EVs) possess the ability to deliver a greater number of mitochondria to recipient brain endothelial cells, thereby augmenting their mitochondrial activity (D Souza et al., 2021). Moreover, Tanveer Ahmad et al. demonstrated that Miro1 (a mitochondrial Rho-gtpase) is able to control mitochondrial migration from MSCs to epithelial cells (ECs), and it can facilitate direct transfer of mitochondria between neurons and other cell types (Ahmad et al., 2014). Nancy Tseng et al. showed that MSCs improved neuronal metabolism after transferring healthy mitochondria to injured neurons *in vitro*, suggesting that artificial transfer of mitochondria to injured cells is a viable way to enhance functional outcomes (Tseng et al., 2021). More and more studies have demonstrated the beneficial effects of mitochondrial supplementation following cerebral ischemia. However, the optimal mode of mitochondria transfer to injured brain tissue remains largely controversial. Further studies are needed to fully elucidate the underlying mechanisms and to assess the safety and efficacy of this treatment (Clemente-Suárez et al., 2023). Consequently, exogenous mitochondrial transfer holds promise as a viable treatment strategy for neurological impairment resulting from cerebral ischemia.

### 3.4 Anti-inflammatory and immunomodulatory effects

Oxidative stress is a crucial mechanism in the pathogenesis of ischemic stroke. Under hypoxic-ischemic conditions, excessive intracellular ROS, such as hydrogen peroxide, hydroxyl radicals and superoxide anions are plenty produced. These ROS can cause oxidative damage to biological components such as proteins, membranes and nucleic acids in brain cells, leading to further deterioration of brain function (Jurcau and Ardelean, 2022). Oxidative stress exerts multiple effects on ischemic stroke. Firstly, oxidative stress can disrupt the redox balance in brain cells, in which excessive accumulation of oxidative substances trigger a cascade of pathological reactions. Secondly, it can cause cerebrovascular injury and inflammation, exacerbating the existing damage. Thirdly, oxidative stress can induce neuronal apoptosis and impair neuronal function, resulting in severe outcomes for ischemic stroke (Sies, 2015; Boltze and Perez-Pinzon, 2022). Given the impact of oxidative stress on ischemic stroke, several preventive and therapeutic strategies have been proposed. Among them, topical application of antioxidants is a practical method. Antioxidants can eliminate intracellular ROS and mitigate brain tissue damage induced by oxidative stress (Pisoschi and Pop, 2015). Yang et al. found that the crebanine, an isoquinoline-related alkaloid, can attenuate brain injury in MCAO rats by inhibiting microglial inflammation and NOX2-mediated oxidative stress (Yang et al., 2023). Guo et al. also found that Lyoniresinol (LNO) exerts neuroprotective effects by activating the PI3K/AKT/GSK-3β/NRF2 signaling pathway to alleviate intracellular oxidative stress (Guo et al., 2023).

Another mechanism through which stem cells work in ischemic stroke is to regulate the activity of the immune system. Glial cells, including oligodendrocytes, astrocytes and microglia are an important part of the peri-infarct microenvironment and are related to the regulation of the immune system after ischemic stroke (Xu et al., 2020) Figure 2. The etiology of ischemic stroke is closely associated with aberrant activation of the immune system and inflammatory response. Following an ischemic stroke, immune cells will play a crucial role in the process of recovery. Firstly, immune cells can orchestrate the inflammatory response. The damage of ischemic stroke is due to ischemia and subsequent reperfusion, triggering an inflammatory cascade. Immune cells such as macrophages, T cells and B cells assume crucial regulatory functions in this process by generating pro-inflammatory factors and cytokines. Secondly, immune cells are indispensable for neuroprotection and post-stroke healing process. Furthermore, these immune cells possess the capability of eliminating neurotoxic substances and cellular debris, concurrently reducing inflammation and apoptosis, both of which contribute significantly to the restoration of brain tissue (Iadecola et al., 2020; Zhang D. et al., 2021; DeLong et al., 2022). The pathological progression of ischemic stroke is primarily mediated by macrophages and microglia. By comprehending the interplay between macrophages and microglia, MHC class II molecules, T cells and neural antigens, as well as their signaling pathways, all the strategies for therapeutic intervention following ischemic events can be identified. In recent years, more and more studies have focused on the role of immune cells in stroke. Targeting the immune cells is a promising therapeutic strategy to improve the prognosis of stroke and to explore immune regulation (Var et al., 2021).

Stem cells can also balance the immune system, which helps prevent excessive activation of immune cells, reduce inflammation by releasing signaling molecules, and improve the long-term prognosis of stroke (Vizoso et al., 2017; Zhou et al., 2019; Liu et al., 2021). MSCs have been found to participate in repairing and maintaining homeostasis by interacting with the inflammatory microenvironment (Wang Y. et al., 2022). Plus, MSCs can regulate the immune response and provide neuroprotective effects by releasing paracrine factors or through EVs, which promote the generation of neurons, oligodendrocytes and astrocytes, and the formation of blood vessel in ischemic brain injury (Dabrowska et al., 2019a; Kim et al., 2020; Tobin et al., 2020). Wang et al. found that recombinant human fibroblast growth factor 21 (rhFGF21) regulates nuclear factor-κb (NF-κB) and peroxisome proliferator-activated receptors (PPARs) signaling pathway to regulate microglia/macrophage-mediated neuroinflammation and tissue repair (Wang et al., 2020).

### 3.5 Regulation of autophagy

It is well acknowledged that autophagy significantly affects cell survival and homeostasis. During ischemic stroke, cells experience hypoxia and insufficient energy, intracellular oxidative over-stress and damage of cellular structure and function. In response, autophagy mechanisms are subsequently triggered to remove damaged organelles and proteins, relieve cellular stress, and promote cellular repair and regeneration (Wang et al., 2018; He et al., 2021a). However, the effect of autophagy in ischemic stroke is biphasic (Rezabakhsh et al., 2022). On the one hand, moderate autophagy removes abnormal proteins and harmful substances through autophagosome-lysosomal degradation, reducing the accumulation of toxic substances and protecting cells from further harm. On the other hand, excessive autophagic activity under ischemic conditions can complicate brain injury (He et al., 2019; He et al., 2021b). Studies have shown that autophagy can be regulated through the mechanistic target of rapamycin (mTOR) pathway (Deleyto-Seldas and Efeyan, 2021). Sun et al. also found that VX-765, a selective small molecule inhibitor of Caspase-1, could upregulate autophagy through the AMPK/mTOR signaling pathway to reduce inflammation and apoptosis after stroke (Sun Z. et al., 2020). Moreover, autophagy modulates cellular energy metabolism and ensures sufficient energy provision to bolster post-stroke recovery (Ajoolabady et al., 2021; Shi et al., 2021; Wang et al., 2021). Yang et al. found that autophagy protects the integrity of the BBB by regulating the redistribution of Claudin 5 (CLDN5) (Yang et al., 2021). In summary, the research progress of autophagy in stroke recovery provides us new ideas and methods, which are expected to bring new breakthroughs in the treatment and rehabilitation of stroke.

### 3.6 Paracrine effects of stem cells

Paracrine functions of stem cells have been shown to ameliorate post-stroke injury and neurological dysfunction (Barzegar et al., 2021) Figure 2. Exosomes are EVs generated by multivesicular bodies, which can facilitate the paracrine effect of stem cells. These exosomes release vesicles containing various biologically active molecules, such as growth factors, proteins, nucleic acids and miRNAs, making them hopeful candidates in treating ischemic stroke (Gurung et al., 2021) Figure 3. By fusing with other cell membranes and releasing their internal bioactive molecules, exosomes can alter the function and activity of target cells. Moreover, by binding to receptors on cells, it initiates signaling pathways in target cells, thereby regulating their function (Colombo et al., 2014; van Niel et al., 2018). Exosomes can not only transmit information and regulate cell behavior, but also have many advantages treating ischemic stroke. First, they can be used to treat patients through intravenous administration or intracranial injection. Studies show that exosomes have low immunity and low risk of vascular obstruction. Due to good tissue permeability and biocompatibility, they can easily pass through the BBB and exert immunomodulatory and neuroprotective effects (Dabrowska et al., 2019a). Second, exosomes can be derived from various cell types, such as MSCs, neural cells and immune cells, thus offering a wide range of options for clinical applications. Finally, exosomes exhibit remarkable stability, enabling their long-term storage under freezing conditions without compromising the biological activity (Gorgens et al., 2022).

**FIGURE 3:**
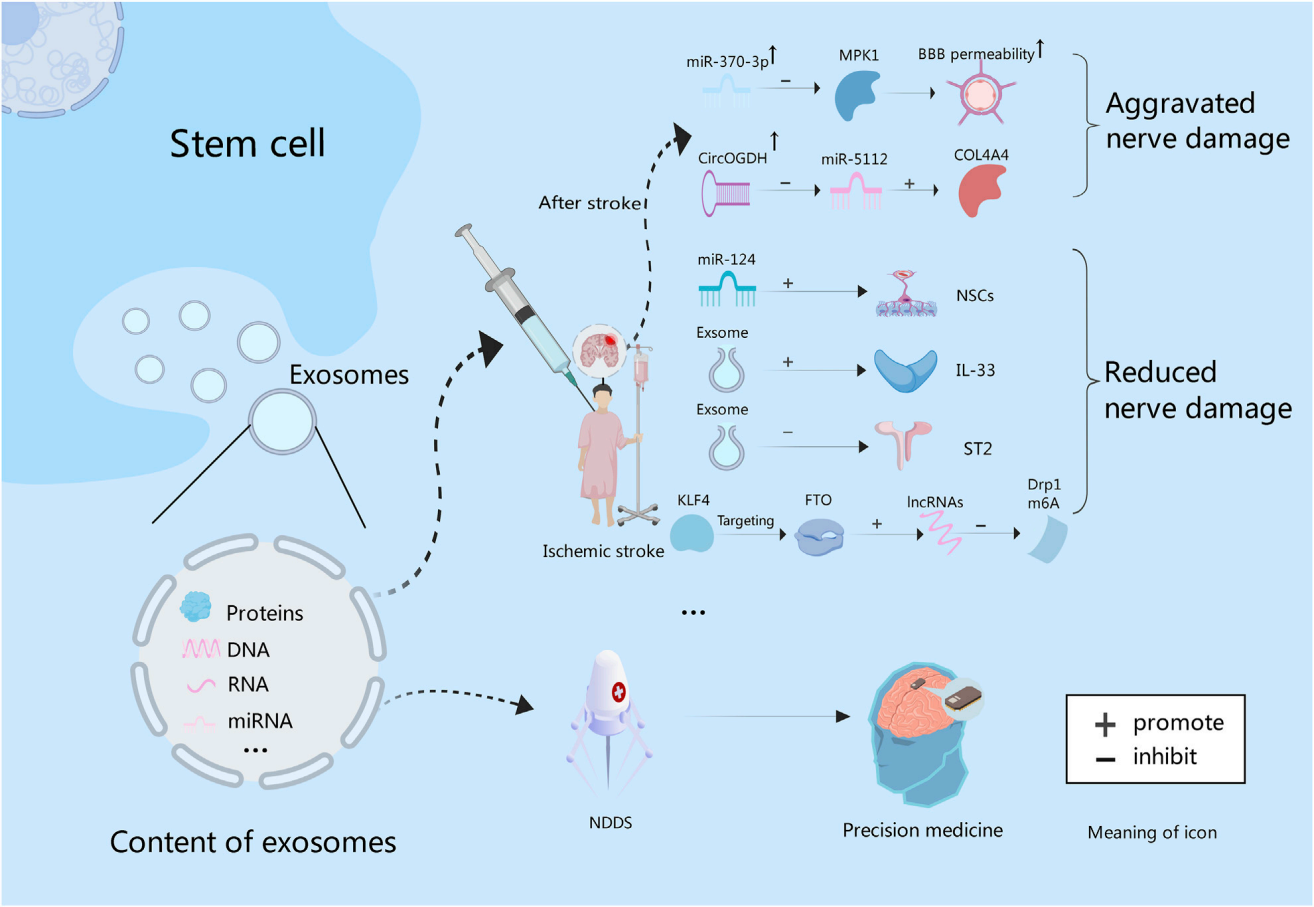
Exosomes contain proteins, and nucleic acids. After stroke, the expression of miR-370 is increased, and BBB permeability can be increased by inhibiting MPK1. While miR-5112 is inhibited by CircOGDH, which can enhance the expression of COL4A4, and thus aggravating brain injury. In addition, exosome-derived miR-124 could promote the proliferation and differentiation of NSCs. Exosomes can improve nerve function by increasing IL-33 level and inhibiting ST2. Also, they can promote lncRNAs expression through KLF4/FTO signaling pathway to inhibit Drp1 m6A modification, thereby inhibiting nerve damage. Finally, exosomes can be used to construct NDDS to deliver targeted drugs to achieve precision treatment of intracranial tumors in the future.

Some experiments have proved that intravascular injection of exosomes can effectively reduce neuroinflammation caused by focal brain injury (Dabrowska et al., 2019b). Liu et al. found that the administration of exosomes derived from BMSCs into a mouse model of ischemic brain injury reduced the volume of cerebral infarction, increased the level of Interleukin-33 (IL-33), inhibited tumorigenicity 2 (ST2), and improved neurological function (Liu et al., 2023). Wang et al. also demonstrated that BMSCs-derived exosomal Kruppel-like factor 4 (KLF4), by targeting obesity-associated protein (FTO), can promote the expression of long noncoding RNAs (lncRNAs) and supress N6-methyladenosine (m6A) modification of dynamin-related protein 1 (Drp1). It thus shrinks mitochondrial dysfunction and neuronal damage in ischemic stroke (Wang Q. S. et al., 2023). The miRNAs, serving as the primary functional executor of exosomes, functions in different stages of stroke and exerts an impact on factors associated with stroke. Theoretically, miRNAs can be valuable tools for stroke diagnosis, prognosis and therapy (Ye et al., 2022). Gu et al. found that miR-370-3p expression rose in stroke rats to increase the permeability of the BBB by inhibiting MPK1. It indicates that MPK1 is the target of miR-370-3p, which can control brain damage after stroke (Gu et al., 2023). Meanwhile, Liu et al. analyzed the interaction between CircOGDH (circular RNA derived from ketoglutarate dehydrogenase) and miR-5112. Inhibition of miR-5112 by CircOGDH was found to enhance the expression of Gallus collagen, type VI, alpha VI (COL4A4), thereby aggravating neuronal injury. This suggests that CircOGDH is a potential therapeutic target for acute ischemic stroke (Liu Y. et al., 2022)Figure 3. In addition, stem cells combined with exosomes also have a significant effect on stroke. An experimental study found that the combined treatment of NSCs and exosomes could significantly improve cerebral infarction in mice by reducing neuronal apoptosis, inhibiting astrocyte overproliferation, and promoting neural remodeling (Zhang R. et al., 2023). In all, exosomes provide a new strategy for the treatment of ischemic stroke.

## 4 Microbiota-gut-brain axis and neuroprotection

With the deepening understanding of the gut microbiome, it has been discovered that there exists a close correlation between the gut microbiota and the nervous system. Targeting the gut and its microbiome offers novel ideas and approaches for treating various nervous system disorders. The microbiota-gut-brain axis is potentially a therapeutic target for safeguarding brain function following stroke injury (Bonsack et al., 2020; Rutsch et al., 2020; Jacobson et al., 2021; Liu L. et al., 2022; Haupt et al., 2023). Ischemic stroke impacts the composition of gut microbiota through neural pathways and hypothalamic-pituitary-adrenal (HPA) pathways; acute brain injury leads to change of intestinal flora and activation of intestinal immune cells, letting proinflammatory cells or bacterial toxins infiltrate the brain tissue through the BBB, which exacerbates cerebral infarction. Conversely, alterations in gut microbiota influence neuroinflammation and post-injury neurological function (Singh et al., 2016; Huang and Xia, 2021; Chidambaram et al., 2022). On top of that, metabolites produced by gut microbiota have a significant impact on the regulation of human immune response, tryptophan metabolism, and serotonin production (Guo et al., 2021; Waclawikova and El Aidy, 2018; O Mahony et al., 2015; Hou et al., 2022). Corinne Benakis et al. found that intestinal dysbiosis can change the immune homeostasis in the small intestine. After stroke, dysbiosis inhibits the trafficking of T cells from the gut to the meninges (Benakis et al., 2016). Moreover, microbial metabolites such as short-chain fatty acids (SCFAs, including acetic acid, butyric acid and propionic acid) modulate immune cells via free fatty acid receptors to regulate immune and inflammatory responses (Pluta et al., 2021). Consequently, it can be adopted as a novel strategy to regulate microbiota balance to influence neurofunction. The microbiota-gut-brain axis can enhance neuroprotection by modulating intestinal microbial community composition and promoting intestinal immune function. Simultaneously, modulation of the neuroendocrine system can improve self-repair capabilities within the nervous system (Bienenstock et al., 2015; Sandhu et al., 2017).

Via understanding and exploiting the microbial-gut-brain axis, we can explore new therapeutic approaches. Certain metabolic products generated by intestinal flora following stroke have been shown to reduce ischemia-reperfusion injury while facilitating neurological functional recovery through inhibition of post-stroke inflammatory responses (Hu W. et al., 2022; Wang T. et al., 2023). Shen et al. found that stroke patients and rats have a large accumulation of branched chain amino acids (BCAAs) due to the imbalance of intestinal flora. Furthermore, BCAAs exacerbate microglia-induced neuroinflammation by activating the AKT/STAT3/NF-κB signaling pathway. It is noteworthy that colonization of the microbiota with *Lactobacillus* helveticus (L.hel) and *Lactobacillus* brevis (L.bre) confers significant neuroprotective activity, especially reducing the buildup of BCAAs (Shen et al., 2023). By modifying dietary habits, increasing probiotic intake, to alter the composition of gut microbiota and its metabolites, it is possible to modulate the gut microbiota community, thereby influencing the production of microbe-derived metabolites such as trimethylamine N-oxide (TMAO) and SCFAs. These metabolites actively participate in maintaining CNS homeostasis, gastrointestinal physiology and immune responses, and they can delay the impairment of intestinal barrier function, reducing the risk of stroke, and promoting post-stroke recovery (Peh et al., 2022). Diet therapy, fecal microbiota transplantation, traditional Chinese medicine therapy or antibiotics have a specific efficacy in regulating intestinal microorganisms and preventing cerebrovascular diseases. However, given that gut microbes are characterized by individual differences and dynamic changes across a range of populations, novel approaches targeting gut homeostasis require further research to refine and standardize the components (Zou et al., 2022). Overall, the theory of the microbiota-gut-brain axis provides us with a novel perspective for comprehending and managing neurological disorders. Despite extensive research on the neuroprotective mechanisms of the microbiota-gut-brain axis, there are still many unanswered questions that need to be further explored. For example, how to accurately assess the functional status of the microbiota-gut-brain axis and how to select appropriate therapeutic strategies to achieve the best outcomes are still under debate. All these issues demand additional investigation and discussion. In the future, we anticipate more studies elucidating the mechanisms underlying the microbiota-gut-brain axis and offering enhanced theoretical foundations and practical guidance for neuroprotection.

## 5 New hope for adjuvant therapy for brain tumor

Stem cell therapy has significant potential for regulating the immune system and holds promise in treating various diseases including brain tumors. However, immunotherapy outcomes in glioblastoma patients have been disappointing due to the low efficacy of drug delivery and insignificant immune response. To achieve successful immunotherapy, efficient drug delivery to the brain and cell targeting are both necessary. Future research will focus on combining stem cells with checkpoint monotherapy and immunomodulatory approaches to develop individualized therapies based on tumor subtypes and metastatic sites (Sampson et al., 2020; Rong et al., 2022). Wang et al. found that CD161 expression is closely related to glioma pathology and molecular pathology, suggesting that it may be a new therapeutic target for glioblastoma. Therefore, it may be feasible to develop CD161 inhibitors and stem cell-derived targeted therapies for the treatment of gliomas (Di et al., 2021). Unfortunately, satisfactory results have not yet been achieved in adult or pediatric brain tumor immunotherapy due to low mutation load and limited immunogenicity along with dormant damage to the CNS during interventions. Further research is needed to explore whether stem cell intervention can enhance the efficacy of brain tumor immunotherapy (Melcher and Kerl, 2021). It is observed that autophagy plays a pivotal role in tumor suppression and cell survival maintenance by engaging in multiple subcellular processes and facilitating interactions between the immune system and tumor cells. Enlightened by this, targeting the regulation of autophagy in stem cell-based tumor therapy holds tremendous promise (Petrosyan et al., 2022). Furthermore, engineered stem cell-derived EVs can be utilized to deliver specific therapeutic substances with treatment-specific functions such as anti-apoptosis, anti-inflammation, immunomodulation, vascular promotion, and neurogenesis (Xiong et al., 2024). The combination of stem cells and exosomes presents a potential multifunctional nano drug delivery system (NDDS) for brain tumor treatment. EVs have high biocompatibility and are not easy to induce immune response, which makes it possible to construct NDDS. Key gene therapy drugs can be delivered to targeted tumor cells through NDDS to achieve precision cancer treatment (Cheng et al., 2021). The experimental results of Cui et al. showed that the construction of NDDS, which can realize the co-delivery of multiple components of traditional Chinese medicine and immune adjuvant, has good BBB penetration ability, and can significantly prolong the median survival time of mice with glioblastoma (Cui et al., 2023). Brain tumors are lethal and strenuous to treat due to various obstacles, including the BBB, the blood-brain tumor barrier (BBTB), overexpression of efflux pumps, infiltrative growth, high heterogeneity, drug resistance mechanisms, immune evasion mediated by the tumor microenvironment (TME), and presence of cancer stem cells (CSCs). Conventional surgical interventions and chemotherapy often fail to achieve satisfactory outcomes. Although advances have been made in combining chemotherapy with other methods, it remains suboptimal to treat brain tumors because of their intricate nature (Yang and Chen, 2022). Combining chemotherapy with stem cell-derived exosomes may hold significant potential as a therapeutic approach. Therefore, further studies on the regulatory effects of stem cells on the immune system and the combination of stem cells and exosomes may provide new strategies for the treatment of brain tumors.

## 6 Stem cell source and delivery challenges

The choosing of a suitable tissue source to get stem cells from is one of the main challenges in stem cell treatment for ischemic stroke. Numerous tissue sources have been investigated, including adult tissue (fat, bone marrow, blood, skin and skeletal muscle), fetal tissue (directly derived from fetal or extra-fetal tissue), embryonic tissue, genetic reprogrammed somatic cells, specifically induced pluripotent stem cells (iPSCs) (Bacakova et al., 2018). Every tissue resource has advantages and disadvantages. For example, BMSCs have been intensively studied and shown encouraging results in preclinical and clinical studies (Chu et al., 2020). Donor-site morbidity, however, may result from the intrusiveness of the isolation procedures. Furthermore, strategies for preserving MSCs’ capacity for self-renewal and differentiation must be developed due to their sluggish growth and differentiation (Brown et al., 2019). Further research is required to determine their potential for differentiation and therapeutic success. Adipose tissue-derived stem cells, on the other hand, maybe readily collected through minimally invasive techniques (Mangin et al., 2019; Khazaei et al., 2022). Thus, to guarantee the best possible outcome for stem cell treatment for ischemic stroke, serious considerations should be taken into the selection of tissue source.

Transporting stem cells to the target region is another difficulty in stem cell treatment for ischemic stroke. Efficient transport is essential for transplanted stem cells to integrate and survive in the ischemic brain successfully. Diverse delivery methods have been investigated, such as intraperitoneal injection, nasal administration, intravenous infusion, intraarterial injection, and intracranial injection (Kasahara et al., 2016; Rodriguez-Frutos et al., 2016; Boncoraglio et al., 2019). Every strategy has its limitations. For instance, intravenous infusion is a safe and practical method of systemic delivery of stem cells, however, it may lead to cell retention and restricted targeting to the ischemic tissue. Intraarterial infusion has more precise targeting but carries the risk of embolism and vascular injury. Although direct injection bypasses the BBB for precise drug delivery, and intracerebral injection exhibits better neurological outcome compared to other routes, it is an invasive procedure that may potentially induce additional tissue damage (Bhatia et al., 2018; Chung et al., 2021; Namestnikova et al., 2021; Fauzi et al., 2023). Therefore, it is essential to develop novel and efficient drug delivery strategies to improve the therapeutic effect of stem cell therapy for ischemic stroke.

Let alone challenges related to tissue sources and delivery, stem-cell therapy for ischemic stroke faces other significant hardness. One hurdle is the immune response triggered by the transplanted stem cells. The host’s immune system can identify these transplanted cells as foreign substances and initiate an immune reaction, which may result in rejection or diminished survival of the transplanted cells. Strategies to overcome this immune response, such as immunosuppressive therapy or immunomodulatory cells, require further exploration (Wang Y. et al., 2022; Achon Buil et al., 2024). The optimal timing and dosage of stem cell transplantation after ischemic stroke remain uncertain, posing a challenge for effective treatment. Early transplantation may disrupt the natural healing process, while delayed transplantation could limit the therapeutic potential of stem cells, timing selection is of importance (Wang et al., 2014; Li et al., 2021). Standardization and assessment of the efficacy of stem-cell therapies are also significant challenges. The risk factors for ischemic stroke are complex. Traditional vascular risk factors (e.g., hypertension, smoking, and obesity) are mostly associated with stroke in older patients. While other risk factors such as illicit drug use, pregnancy, arterial dissection, and patent foramen ovale (PFO) are associated with stroke at younger ages (Ekker et al., 2018; Wang Y. J. et al., 2022). This has brought great difficulties to the standardization of stem cell therapy. The effect of stem-cell therapy is influenced by chronobiological mechanisms, drug-therapy interactions, patient age, and coexisting conditions. Adequate budgets and models are needed to validate all these relevant factors and combinations of them in preclinical studies (Boltze et al., 2017; Boltze et al., 2019). Finally, although early clinical trials have reported that stem cell therapy is safe and feasible, long-term effects, such as the risk of late complications and cancer-causing possibility are uncertain. Long-term follow-up studies are essential to assess the safety of stem cell therapy in stroke patients and to monitor potential long-term effects. Based on the evidence, further research is needed to upgrade the overall efficacy of stem cell therapy for ischemic stroke.

## 7 Conclusion

The high disability and mortality rate of ischemic stroke makes it a serious threat to human health. At present, there is no ideal strategy for neurological protection after stroke except acute thrombolysis and endovascular intervention. Stem cells have the capability of self-renewal and multi-directional differentiation, as well as the biological characteristics of rapid proliferation, easy to culture and low immunogenicity. Current studies have shown the potential of stem cells in promoting nerve repair and angiogenesis, restoring mitochondrial function, regulating autophagy, alleviating oxidative stress and immunomodulation. At the same time, the combination of stem cells and exosomes is a promising treatment strategy for ischemic stroke. In addition, the microbiota-gut-brain axis opens a new perspective for us to understand and treat neurological diseases. However, the potential of stem cells in the treatment of ischemic stroke and other neurological diseases still needs to be explored to improve the efficacy and implement clinical application.
